# Conformational variability of cyanobacterial ChlI, the AAA+ motor of magnesium chelatase involved in chlorophyll biosynthesis

**DOI:** 10.1128/mbio.01893-23

**Published:** 2023-09-22

**Authors:** Dmitry Shvarev, Alischa Ira Scholz, Arne Moeller

**Affiliations:** 1 Structural Biology Section, Department of Biology/Chemistry, Osnabrück University, Osnabrück, Lower Saxony, Germany; 2 Center of Cellular Nanoanalytics Osnabrück (CellNanOs), Osnabrück University, Osnabrück, Germany; Case Western Reserve University School of Medicine, Cleveland, Ohio, USA

**Keywords:** magnesium chelatase, chlorophyll biosynthesis, AAA+ protein, ChlI motor subunit, photosynthesis, cryo-EM, *Nostoc *sp. PCC 7120, cyanobacteria

## Abstract

Magnesium chelatase is a conserved enzyme complex responsible for the first committed step of chlorophyll biosynthesis in photosynthetic organisms, which is the addition of magnesium to the chlorophyll precursor, protoporphyrin IX. The complex is composed of the catalytic subunit ChlH, the bridging subunit ChlD, and the subunit ChlI, which serves as the motor that drives the entire complex. Although the enzyme is well-characterized functionally, high-resolution structures are available only for individual subunits. Hence, the full assembly and molecular mechanism of the enzyme complex remain unknown. Here, we used cryogenic electron microscopy, supported by biochemical analysis and mass photometry, to determine the structures of the ChlI motor subunit of magnesium chelatase under turnover conditions in the presence of ATP. Our data reveal the molecular details of ChlI oligomerization and conformational dynamics upon ATP binding and hydrolysis. These findings provide new insights into the mechanistic function of ChlI and its implications for the entire magnesium chelatase complex machinery.

**Importance:**

Photosynthesis is an essential life process that relies on chlorophyll. In photosynthetic organisms, chlorophyll synthesis involves multiple steps and depends on magnesium chelatase. This enzyme complex is responsible for inserting magnesium into the chlorophyll precursor, but the molecular mechanism of this process is not fully understood. By using cryogenic electron microscopy and conducting functional analyses, we have discovered that the motor subunit ChlI of magnesium chelatase undergoes conformational changes in the presence of ATP. Our findings offer new insights into how energy is transferred from ChlI to the other components of magnesium chelatase. This information significantly contributes to our understanding of the initial step in chlorophyll biosynthesis and lays the foundation for future studies on the entire process of chlorophyll production.

## INTRODUCTION

Photosynthesis is a vital process in plants, phototrophic bacteria, and cyanobacteria whereby light energy is absorbed and used to synthesize organic molecules. Essential for photosynthesis are chlorophylls, tetrapyrrole compounds that harvest the energy of photons. The first committed step in chlorophyll biosynthesis is the insertion of Mg^2+^ into the macrocycle of the chlorophyll precursor protoporphyrin IX (1 – 3), performed by the magnesium chelatase enzyme complex. Magnesium chelatase is fueled by ATP and consists of three subunits: ChlH, ChlD, and ChlI. The motor subunit ChlI belongs to the superfamily of AAA+ (ATPases associated with diverse cellular activities) proteins and, through binding and hydrolyzing of ATP, provides the energy for the thermodynamically unfavorable reaction of Mg^2+^ insertion into protoporphyrin IX (4, 5). Magnesium chelatase has been extensively characterized functionally (6, 7), including its activity (5, 8, 9), substrate specificity (10, 11), and subunit interactions (8, 12 – 14). However, how the subunits cooperate and mechanistically contribute to the process of magnesium chelation remains elusive.

Several structures of magnesium chelatase components have recently been reported and provided insights into the molecular organization of the enzyme (15 – 17). The monomeric structure of the AAA+ protein BchI, the ChlI homolog from the photosynthetic bacterium *Rhodobacter capsulatus* (hereafter, *Rhodobacter*), revealed that the protein has an unusual domain arrangement compared to other AAA+ proteins (15), placing BchI/ChlI in clade 7 of AAA+ proteins (18). Typically, AAA+ proteins consist of two subdomains, the N-terminal large and the C-terminal small. The large subdomain contains a five-strand β-sheet, the conserved Walker A and Walker B motifs, and additional key residues responsible for ATP binding and hydrolysis. The α-helical small subdomain follows the large subdomain, covering the nucleotide-binding pocket and contributing to the oligomerization of the protein. In addition, different AAA+ proteins can carry specific structured inserts introduced into conserved large and small subdomains and mediate distinct functions of these proteins (19). As such, the C-terminal domain (small AAA+ subdomain) of BchI is dislocated backward from the nucleotide-binding site in contrast to other AAA+ proteins, where it tends to face upward. In addition, the BchI protein carries three structured insertions, i1, i2, and i3, located before β-strand β2, within the α-helix α3, and after the α-helix α4. Insertions i2 and i3 can be classified as H2 and PS1 inserts, respectively (19). Of note, the insertion i1 is specific for BchI and may play a role in interactions with other components of the magnesium chelatase complex (15).

Like other members of the AAA+ superfamily, ChlI/BchI forms oligomers in solution, usually hexamers (15, 16, 20). Recently, the structure of the ChlI hexamer from the cyanobacterium *Synechocystis* sp. PCC 6803 (hereafter, *Synechocystis*) was solved by X-ray crystallography (16). In that structure, monomers exhibited well-structured AAA+ large and small subdomains, while insertions i1-i3 were not observed. No nucleotides were bound in the protein, but the ATP-binding pockets at the intermonomer interface showed a typical arrangement for AAA+ proteins.

Moreover, ChlI/BchI forms oligomeric complexes with the other component of magnesium chelatase, ChlD/BchD (8, 12, 21). Previous cryogenic electron microscopy (cryo-EM) studies, at intermediate resolution, demonstrated that the BchID subcomplex of magnesium chelatase exhibits a double-ring architecture and undergoes conformational changes upon incubation with different nucleotides (20, 22). However, no ATPase activity has been detected for ChlD/BchD, despite belonging to the AAA+ superfamily (8, 21). Apparently, ChlD/BchD only links ChlI/BchI to the porphyrin-binding ChlH/BchH, the third subunit of the complex, leaving the motor function exclusively to ChlI/BchI. Recent biochemical data confirmed that ChlD bridges the ChlI motor to ChlH (13). In conjunction, the structural and biochemical data suggest that the ChlI/BchI and ChlD/ChlI rings stack on top of each other, and that this assembly hub powers the ChlH/BchH catalytic component of the complex, but how the energy is transferred from ChlI/BchI during this process is unknown.

Here, we used a combination of cryo-EM and functional analysis to investigate the mechanism of the ChlI motor subunit of magnesium chelatase from the cyanobacterium *Nostoc* sp. PCC 7120 (hereafter, *Nostoc*). We examined the oligomerization of ChlI using mass photometry (23) and determined the structures of different ChlI oligomeric states with cryo-EM. Our data provide insights into the formation and function of the ChlI hexameric complex, highlighting the ATP-induced conformational changes of the protein. Our results suggest a mechanism whereby the ChlI insertions may facilitate the transfer of ATP binding and hydrolysis energy to the other components of the magnesium chelatase complex.

## RESULTS

### Purification and biochemical characterization of *Nostoc* ChlI

**FIG 1:**
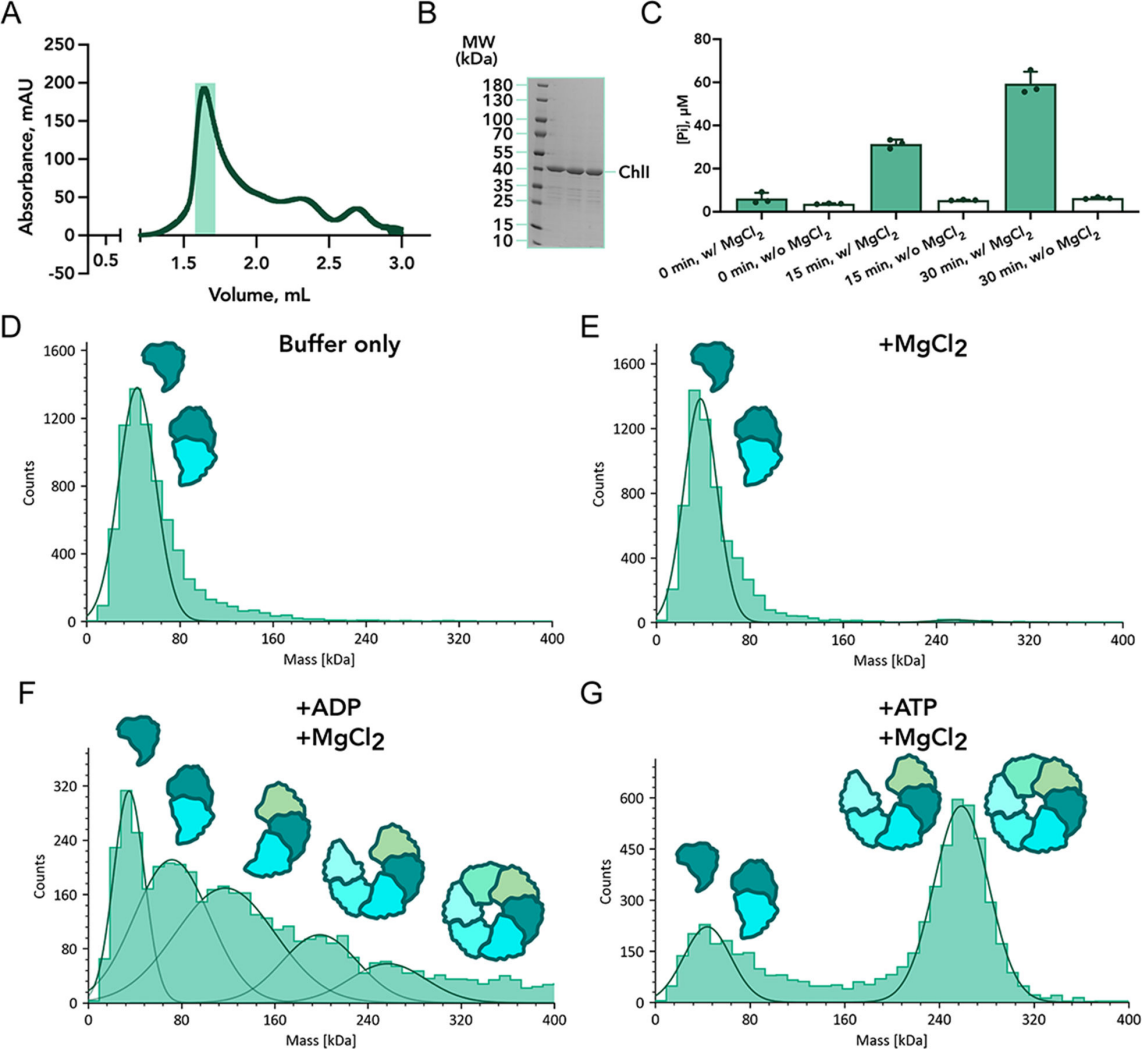
Biochemical and oligomerization analysis of purified ChlI. (**A**) ChlI SEC profile and peak fractions are indicated by a teal rectangle. (**B**) Corresponding SDS electrophoresis gel of SEC peak fractions. (**C**) Phosphate release from ATP hydrolysis by ChlI (12.2 µM) measured using the malachite green assay. (**D–G**) ChlI oligomeric state analyzed in Tris buffer alone (**D**), with the addition of MgCl_2_ (50 mM) (**E**), with the addition of ADP (10 mM) and MgCl_2_ (50 mM) (**F**), or with the addition of ATP (10 mM) and MgCl_2_ (50 mM) (**G**). For mass photometry, representative results of three independent experiments are shown.

We overexpressed the recombinant *Nostoc* ChlI subunit (encoded by the *all0152* gene) in *Escherichia coli* and purified it by affinity and size-exclusion chromatography (Fig. 1A). The size-exclusion chromatography profile reveals a single major peak containing pure ChlI protein of approximately 40 kDa, as shown by SDS gel electrophoresis (Fig. 1B). We confirmed that ChlI performs ATP hydrolysis in the presence of MgCl_2_, as expected (Fig. 1C).

As shown by mass photometry, the protein is mainly monomeric or spontaneously forms small oligomers in solution (Fig. 1D and E), which are primarily dimers and are inaccessible by cryo-EM. Therefore, we stimulated the formation of hexamers by incubating the ChlI protein with ATP and MgCl_2_, similar to previous reports on the bacterial ortholog of ChlI, named BchI, and other AAA+ proteins (15, 24, 25). Mass photometry discovered two significant populations of ChlI oligomers that correspond to mono-/dimers and penta-/hexamers in the presence of ATP and MgCl_2_ (Fig. 1G). Interestingly, when we added ADP instead of ATP, we observed a wide range of mass distribution in the sample (Fig. 1F). We interpret this as a variety of ChlI oligomeric species, from monomers to hexamers, but with a much smaller fraction of large oligomers.

### Cryo-EM structures of hexameric ChlI

To elucidate the molecular mechanism by which ChlI drives the entire magnesium chelatase complex, we determined the protein structures in the presence of ATP and MgCl_2_ using cryo-EM. For our study, we collected and combined two data sets of approximately 4,000 movies each (Fig. S1 and S2A) and processed the data using cryoSPARC (26). In support of our biochemical data, we mainly observed the ChlI hexamers in the cryo-EM sample (Fig. S2B). DTT (27) and LMNG were added to combat the otherwise strong preferred particle orientation toward the top/bottom views.

**FIG 2:**
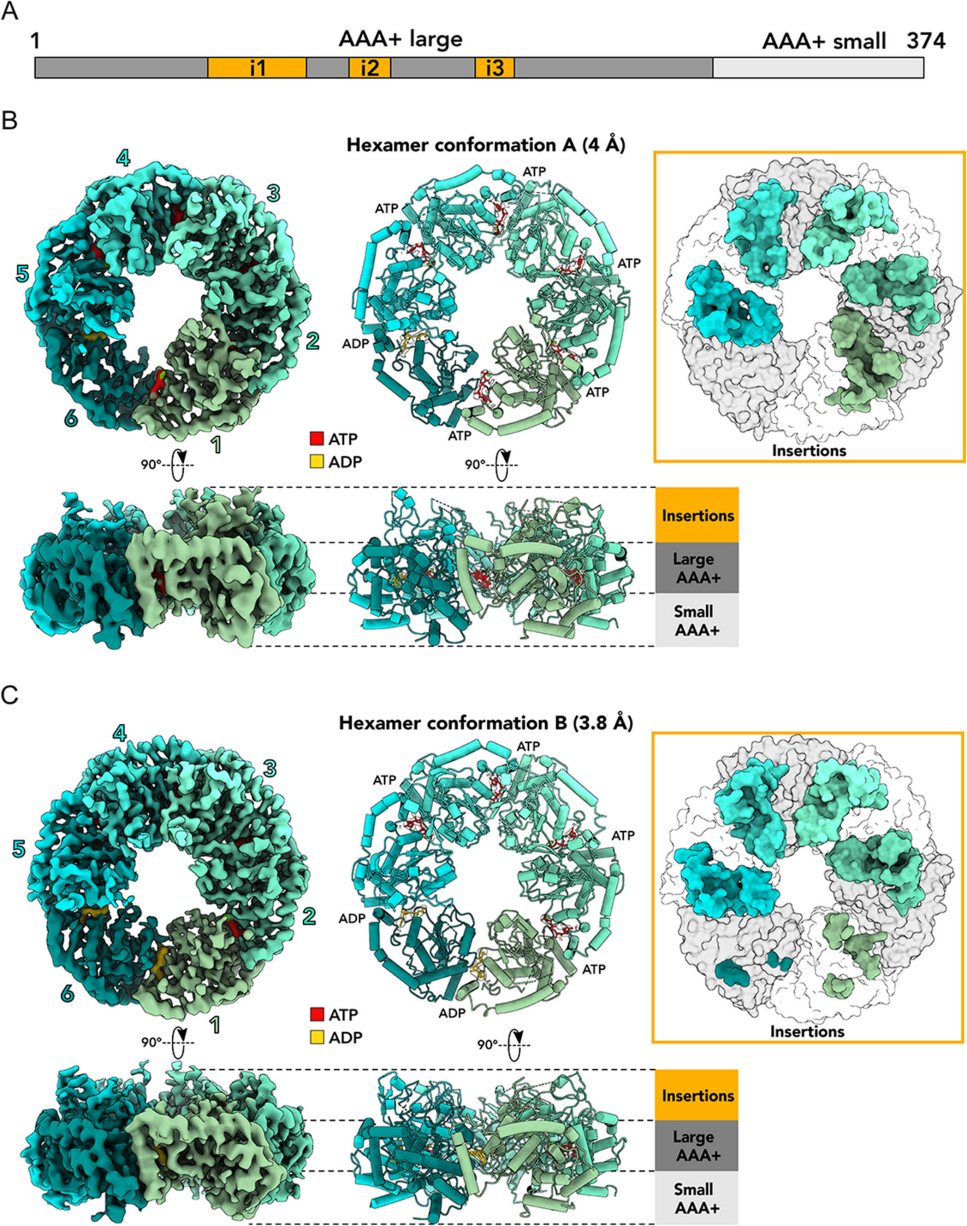
Cryo-EM analysis of ChlI. (**A**) Schematic overview of the ChlI protein sequence with AAA+ subdomains and characteristic insertions i1–i3 indicated. (**B and C**) Cryo-EM maps (left) and corresponding ribbon representations (center) of ChlI hexamer structures in conformations A (B) and B (C) shown as top and side views. The right panels in panels **B** and **C** show top views for each hexamer conformation as surface representations with insertions colored by subunit. Densities of modeled nucleotides are shown within the ChlI structure ribbon representations. Large and small AAA+ subdomains and insertions are indicated in the side views of the structures.

**FIG 3:**
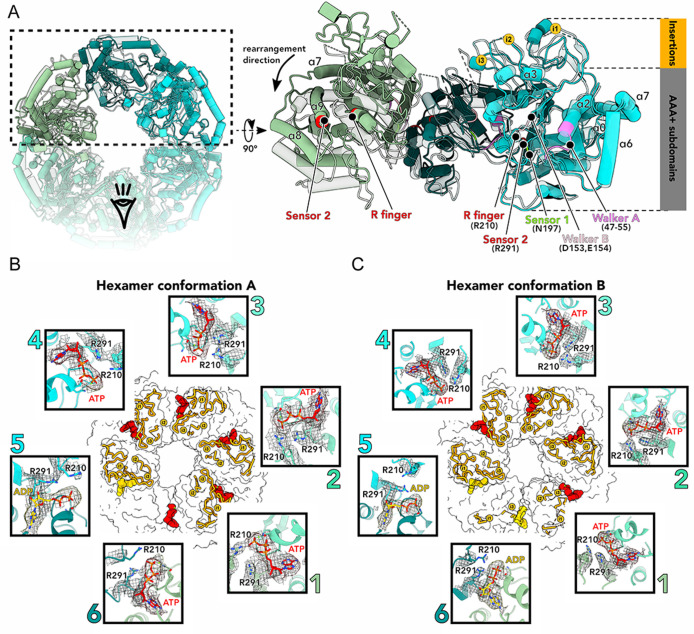
The conformations of ChlI hexamers depend on the nucleotides bound. (**A**) Top view (left panel) and side view from the central pore of the aligned ChlI hexamer conformations A (colored) and B (semi-transparent) in ribbon representation. Monomers are colored as in Fig. 2. The positions of structured insertions i1-i3 and AAA+ subdomains are indicated on the right. Key motifs and residues and several α-helices are marked on the structure for orientation. (**B and C**) Molecular surface representations of conformation A (**B**) and conformation B (**C**). Bound ATP (red) and ADP (yellow) molecules are shown as sphere representations inside the hexamers, and structured inserts are shown as ribbons (orange). Insets show close-up views of the nucleotide molecules with surrounding structural elements of ChlI monomers (numbered and colored as in Fig. 2). The associated cryo-EM densities are zoned around the molecular models of nucleotides and residues 210 and 290–292 and are shown as a mesh. The viewing contour levels of the densities shown are 0.094 (**B**) and 0.12 (**C**). The residues of Walker A, the arginine finger R210, and the sensor 2 R291 are indicated.

Iterative rounds of 3D classification, performed by multiple-class *ab initio* reconstructions followed by heterogeneous refinements (Fig. S1), revealed two distinct classes, which we named hexamer conformations A and B. These reconstructions achieved global resolutions of 4 Å and 3.8 Å, respectively, with much greater local resolutions in the central parts of the structures (Fig. S2). The resulting cryo-EM maps allowed for accurate molecular model building and assignment of nucleotides to their respective densities (Fig. 2B, C and 3; Movie S1 and Movie S2). Interestingly, each monomer contains a bound nucleotide molecule (ATP or ADP) in both hexameric states. The *Nostoc* ChlI hexameric ring conformations have diameters of approximately 114–117 Å (Fig. S3A), with a central pore of about 33 Å. Our reconstructions with no symmetry imposed revealed structural differences between the constituent monomers within the ring. Notably, the region of the insertions displayed varying levels of structural organization among the monomers (Fig. 2B and C, right panels). This observation differentiates our structures from the previously published structure of *Synechocystis* ChlI (16), which did not resolve any structured insertions (Fig. S3). Furthermore, in our ChlI hexamers, the monomers are pulled together in the upper region where the insertions are located, making the *Nostoc* ChlI ring more compact in contrast to *Synechocystis* (Fig. S3B and C). Comparisons of ChlI monomers from our hexameric structures with ChlI monomers from the *Synechocystis* hexamer revealed no major structural differences as indicated by the calculated RMSD values, except for the presence of structured insertions in our structures (Fig. S3D and E). However, the less well-aligned regions that are essential for ATP hydrolysis (Walker A, Walker B, sensor 1 motifs) (Fig. S3D and E) suggest that our ChlI structures represent rather different conformational states of the ChlI activity cycle than the *Synechocystis* structure.

The core of each *Nostoc* ChlI monomer consists of the large and small AAA+ subdomains, resembling a commonly found AAA+ fold (19, 28). Like in *Rhodobacter* BchI (15, 20) and *Synechocystis* ChlI (16), the *Nostoc* ChlI N-terminal large subdomain consists of six α-helices, starting with the N-terminal α-helix α0, and five β-strands, and the C-terminal small subdomain consists of four additional α-helices (Fig. 3A). The small AAA+ subdomain, also called the lid domain, is connected to the large subdomain by α-helices α6 and α7, which can also be considered the presensor two insert (19), thus assigning ChlI to clade 7 of AAA+ proteins.

ChlI and its close homologs (15) contain the structured insertions i1, i2, and i3 (Fig. 2 and 3; Fig. S3) in addition to the standard AAA+ subdomains. Insertion i2 interrupts helix α3 in its first half, and i3 starts immediately after helix α4. Therefore, according to their positions, hairpin insertions i2 and i3 can be attributed to the H2 and PS1 inserts of other AAA+ proteins (19). The presence of these insertions further supports the relation of ChlI to clade 7 of AAA+ proteins. On the other hand, the relatively large insert i1 is located immediately after α2 and is more specific for ChlI-like proteins.

### Conformational changes of ChlI upon ATP hydrolysis

We attribute our hexamer structures of ChlI (Fig. 2 and 3; Movie S1 and Movie S2) to its two different states throughout the ATP hydrolysis cycle. Conformation A is characterized by ATP molecules bound in five monomers and ADP in one (Fig. 3B), while conformation B has ATP bound in four monomers and ADP in two (Fig. 3C). The nucleotide-binding pockets at the interfaces of the monomers demonstrate a standard architecture, including the Walker A and Walker B motifs. Arginine finger R210 and sensor 2 R291 residues from the adjacent monomer additionally coordinate the nucleotide molecule (Fig. 3) and contribute to the inter-subunit cooperation within the hexamer, which is typical for AAA+ proteins (29, 30). In the hexamer structures, the nucleotide state of each monomer is related to the structural organization of the insertions of that monomer, such that when an ATP molecule is bound, the insertions are folded and in an upward position (Fig. 2 and 3). If ADP is bound, the inserts of this monomer are more flexible or even unfold, as demonstrated by fuzzy or absent corresponding cryo-EM densities (Fig. 2). Interestingly, in the observed ADP-bound states, the side chains of the adjacent R210 arginine fingers are positioned somewhat away from the nucleotide (Fig. 3B and C, inset 5), in line with data from other AAA+ proteins (30). In contrast, in most ATP-bound states in our maps, these side chains are oriented toward the nucleotide (Fig. 3B and C, insets 1–3). This may indicate that these residues also contribute to the coupling of ATP hydrolysis with the reorganization of the inserts in ChlI. Nevertheless, we cannot completely exclude the possibility that the nucleotide densities also correspond to mixtures of nucleotide states, for example, the density at monomer five in hexamer conformation A (Fig. 3B).

Not only the structured inserts undergo conformational changes upon ATP hydrolysis in ChlI but also the whole hexamer ring experiences rigid-body rearrangements apparently caused by the ATP hydrolysis-induced reorganization of key amino-acid residues in close proximity to the nucleotide-binding pocket (Fig. 3A; Movie S3). Thus, the above-mentioned changes in the positions of the R210 arginine finger and R291 sensor 2 side chains might affect the interface with the adjacent monomer’s α-helix α6, thereby initiating the overall reorganization of the entire ring and of the structured inserts. Moreover, the conformational rearrangements within the hexamer might be additionally mediated through the interfaces between insertion i1 and α-helices α6-α7 of the adjacent monomer and insertion i3 and adjacent monomer’s α-helix α3 connected to insertion i2.

### Pentameric structure of ChlI

**FIG 4:**
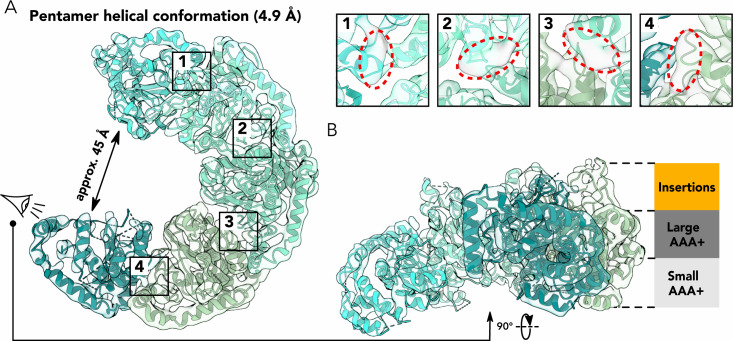
Pentameric spring-washer-like structure of ChlI. (**A**) Top view of the cryo-EM density (semi-transparent) and corresponding molecular model in ribbon representation colored by subunit as in Fig. 2. Numbered insets on the right show close-up views of densities corresponding to bound nucleotide molecules (colored gray and highlighted by red dotted ovals). (**B**) Helical ChlI pentamer viewed from the side; positions of the insertions (orange) and AAA+ subdomains (gray) are indicated.

Our 3D classifications revealed a class corresponding to a pentameric structure of ChlI (Fig. 4), which we attribute to an intermediate state of ChlI complex formation. The map reached an overall resolution of 4.9 Å and differs significantly from the hexamer maps (Fig. 2 and 3). The ChlI pentamer shows a spring-washer-like helical organization with an opening of approximately 45 Å between the first and last monomers in the complex, which would be sufficient to accommodate an additional monomer of ChlI. The resolution obtained for this structure allowed for the fitting of α-helices and β-strands of ChlI monomers but restricted the accurate determination of bound nucleotide molecules. However, the cryo-EM density obtained contains areas that are not occupied by the polypeptide chain of the ChlI protein and are close to the nucleotide binding pocket (Fig. 4A, insets 1–4). Therefore, we suggest that the ChlI pentamer carries at least four nucleotide molecules that we, however, could not unambiguously assign to ATP or ADP. The three central monomers of the pentamer contain partially folded insertions (Fig. 4B) and generally show higher local resolution (Fig. S2C). In contrast, the two peripheral monomers are less resolved, possibly due to the increased flexibility of the ChlI pentamer at its ends.

## DISCUSSION

In this study, we analyzed the ChlI motor subunit of the magnesium chelatase from the filamentous nitrogen-fixing cyanobacterium *Nostoc* sp. PCC 7120 (31, 32). Using biochemical methods and cryo-EM, we discovered two asymmetric hexameric states and one pentameric state of ChlI in the presence of ATP (Fig. 1 to 4). Mass photometry showed that ATP induces the formation of large ChlI oligomers such as hexamers and pentamers (Fig. 1G). This is in line with previous reports on the ATP-dependent oligomerization of ChlI homologs (20, 25) and the disruption of protein hexamerization caused by mutations in key amino-acid residues involved in the ATPase activity of ChlI homolog BchI (33). Of note, our pentameric structure of ChlI also contains nucleotide molecules bound between adjacent monomers (Fig. 4A), supporting the model that ChlI requires ATP for oligomerization. Interestingly, adding ADP also stimulates oligomer formation by ChlI, but it is insufficient for stable hexamer formation (Fig. 1F).

The ChlI monomers in our structures show similarities to other AAA+ proteins and previously characterized ChlI-like proteins (19, 28 – 30), including BchI from *Rhodobacter* (15) and ChlI from *Synechocystis* (16) (Fig. S3). However, our hexameric ChlI structures exhibit unique variations in the architecture of the insertion domains, dependent on the nucleotide state of the monomers, which were not observed previously in close homologs of ChlI. The insertions are folded upward when a monomer is ATP-bound and are more flexible or disordered when ADP is bound (Fig. 2 and 3). It has been shown for the AAA+ proteins dynein and CbbQO-type Rubisco activase that their H2 and PSI inserts have an essential mechanistic function since they bind to partner proteins and couple this with ATP hydrolysis (34 – 36). Similarly, we suggest that the alternate appearance of insertions i1–i3 in ChlI may be essential for interactions with other subunits and thus for the mechanism of magnesium chelatase.

**FIG 5:**
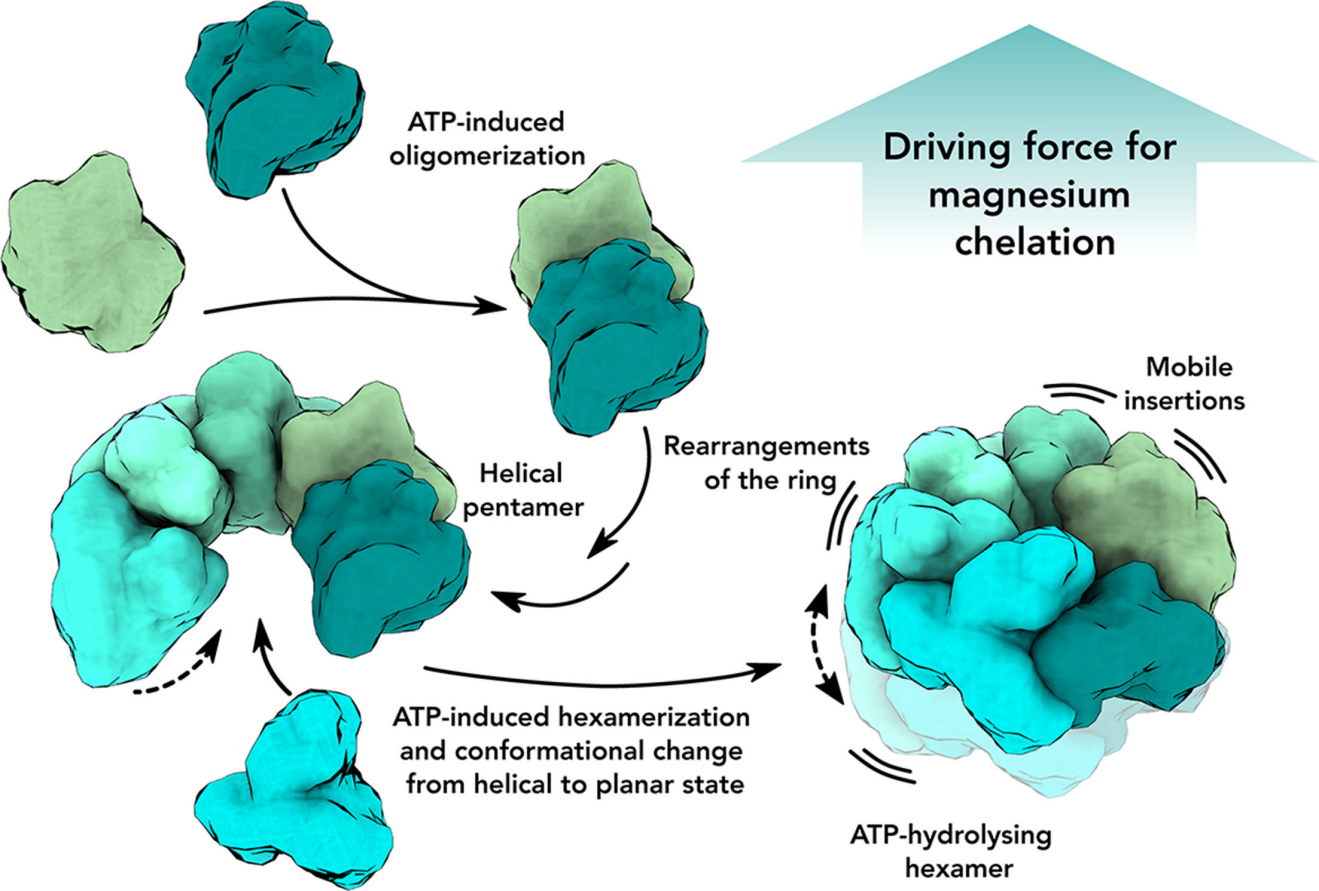
Model of ChlI oligomerization and mechanism. In the presence of ATP, ChlI monomers begin to oligomerize and eventually form a stable spring-washer-like helical pentameric structure. Once the sixth monomer and the sixth molecule of ATP bind to the pentamer, the hexamer ring is formed, and ATP hydrolysis is initiated. The ATP hydrolysis cycle, which proceeds from one monomer to the next, increases the flexibility of the insertions and forces the ChlI ring from a helical to a planar conformation. The conformational changes in the insertions and the entire ChlI ring provide the driving force for the functioning of the whole magnesium chelatase complex.

Based on our data, we propose a sequence of events that ChlI undergoes in the presence of ATP (Fig. 5). First, ChlI monomers interact to form small oligomers upon ATP binding or spontaneously (Fig. 1D and E). The oligomers then grow and form helical spring-washer-like pentameric structures, potentially representing a resting state of the enzyme (Fig. 1G and 4), similar to the Lon protease’s open helical state (37) or the AAA+ protein RavA, which creates a mix of open spiral and closed planar ring state populations (38). Upon binding another monomer to the pentamer, ChlI undergoes rigid-body rearrangements to form a nearly planar hexameric ring. This addition of the sixth monomer to the complex would then initiate ATP hydrolysis, as proposed for Vps4 (39).

According to our model, ATP hydrolysis occurs in one monomer at a time, triggering rearrangements involving residues near the nucleotide-binding pocket. This induces reorganization of the monomer’s insertions and flattening of the entire hexamer (Fig. 2 and 3; Movie S3), which may contribute to ATP hydrolysis in the adjacent monomer, similar to the mechanism of peptide-translocating AAA+ proteins (28). We speculate that after all the monomers have converted ATP to ADP, the ring disassembles for nucleotide exchange.

The architecture and mechanism of full magnesium chelatase remain open questions. It has been proposed that ChlI forms a two-tiered ring complex after binding to ChlD (15, 16), similar to the type II AAA+ proteins (28). Moreover, the corresponding low-resolution structure of the homologous BchI-BchD double-ring complex has been determined (20). On the other hand, biochemical data show that the arginine finger and sensor 2 residues, located at the intermonomer interface, and the entire AAA+ module of ChlD are essential for binding to ChlI in *Synechocystis* (12). This may indicate that ChlD and ChlI form a mixed hetero-hexameric one-tiered ring complex. We have performed AlphaFold (40, 41) modeling of complex formation between the monomers of ChlI and ChlD from *Nostoc* and show in our prediction that the ChlD monomer has an almost identical interface with ChlI as the interface between ChlI monomers in our cryo-EM structures (Fig. S4). This observation may support the idea that ChlI and ChlD form a mixed ring within magnesium chelatase.

In sum, after the oligomerization of ChlI with ChlD, protophyrphyrin IX-bound ChlH would dock to the complex and serve as a substrate for the ChlI-ChlD subcomplex. Then, the ATP-induced conformational changes within ChlI-ChlD would transmit the power stroke to ChlH, which would catalyze the insertion of magnesium ions into protophyrphyrin IX. It is suggested that the unique inserts of ChlI are responsible for interactions with ChlD and ChlH (14, 19). Therefore, the conformational changes of the inserts, in combination with other ATP-induced structural rearrangements that we discovered within the ChlI ring (Fig. 2 and 3; Movie S3), may contribute to the energy transmission to the catalytic subunit ChlH. Alternatively, these specific insertions may play a role in the activity regulation of ChlI, similarly to the corresponding intermediate domains in the AAA+ protein HslU (42, 43). We can speculate that such regulation of the entire magnesium chelatase activity may also be achieved through interactions of the ChlI insertions with the Mg^2+^-binding integrin domain or the linker region of ChlD (Fig. S4), which are shown to be essential for the Mg-chelatase activity and interactions between the subunits (44, 45). To conclude, our data provide a mechanistic insight into the mechanism of ATP-driven activity of the ChlI motor protein. Our data also pave the way for future structural studies of whole magnesium chelatase, which are needed to reveal the exact subunit interfaces and the mode of energy transduction within the complex.

## MATERIALS AND METHODS

### Protein overproduction and purification from *Escherichia coli*

The gene *all015*2 was amplified from the genomic DNA of *Nostoc* sp. PCC 7120, using primers purchased from Biolegio with the sequences 5′-ATT TTG TTT AAC TTT AAG AAG GAG ATA TAC ATG CAT CAT CAT CAT CAT CAC ACT CCA ACA GCT CAA ACC ACG G-3′ and 5′-GTG ATG ATG ATG ATG ATG GCT GCT GCC CAT GTT ACC GGA CAC CTG TCT TAA TTT G-3′, and was cloned using Gibson assembly (46) into plasmid pET15b (Novagen/Merck), which was digested with NcoI. The N-terminal His-tag was introduced into the sequence. The protein was overproduced in *E. coli* LOBSTR cells (47). The cells were grown in Luria broth medium at 37°C with continuous shaking. Protein synthesis was induced by adding 0.1 mM isopropyl-β-d-thiogalactoside at an OD_600_ of 0.6 and incubating the cultures overnight at 18°C with shaking. After induction, cells were harvested by centrifugation at 5,000 × *g* for 20 min at 4°C. The pellet was resuspended in Tris buffer (20 mM Tris, 200 mM NaCl, pH 7.5) containing 1 mM phenylmethylsulfonyl fluoride and 1 mg/mL lysozyme and incubated at 4°C for 1–2 hours with shaking. Then, 1 mg/mL of DNAse I and 5 mM MgCl_2_ were added, and the suspension was incubated for another 1–2 hours. The solutions were centrifuged at 17,000 × *g* for 30 min at 4°C. The supernatant containing extracted soluble proteins was used to purify recombinant ChlI by affinity chromatography using a gravity flow column with nickel-nitriloacetic acid resin (Thermo Scientific). The column was equilibrated with 10 column volumes of Tris buffer (see above) containing 10 mM imidazole. The lysate was applied and incubated on the column for 2 hours, followed by washing with Tris buffer containing 20 mM imidazole, and ChlI was eluted with Tris buffer containing 250 mM imidazole. The concentrated eluate was applied to a Superdex 200 Increase 15/150 Gl column (Cytiva) for size-exclusion chromatography (SEC) and eluted in 50 µL fractions using an ÄKTAgo purification system (Cytiva). Peak fractions were used for further analysis.

### ATP hydrolysis assays

The ATP hydrolysis activity of ChlI was assessed by quantification of free orthophosphate using the malachite green phosphate assay kit (Sigma-Aldrich) according to the manufacturer’s protocol. Briefly, ChlI at 0.5 mg/mL (12.2 µM) was incubated in Tris buffer (see above) with 10 mM of ATP and with or without the addition of 50 mM MgCl_2_ for different time intervals at 37°C. After incubation, the samples were diluted until the ATP concentration reached 0.25 mM and immediately frozen in liquid nitrogen to stop the reaction. The samples were then thawed, transferred to a 96-well plate, and treated with the malachite green reagent. The plate was incubated for an additional 30 min at room temperature for color development, and absorbance at 620 nm was measured using a SpectraMax M3 plate reader (Molecular Devices). Phosphate concentrations in the samples were determined from a previously generated standard curve.

### Mass photometry

Mass photometry (23) experiments were performed using a Refeyn TwoMP instrument (Refeyn Ltd.). Data were obtained using AcquireMP software and analyzed using DiscoverMP (both Refeyn Ltd.). Glass coverslips were used for sample analysis. Perforated silicone gaskets were placed on the coverslips to form wells for each sample to be measured. ChlI samples were incubated at a concentration of 0.5 mg/mL in Tris buffer (see above) with various additives (Fig. 1D through G). Samples were further diluted 1:5 immediately prior to analysis, and 2 µL of this solution was added to 18 µL of Tris buffer placed in the gasket for the measurement.

### Cryo-EM sample preparation and data acquisition

Prior to cryo-EM, ChlI samples were examined by negative-stain electron microscopy using a 2% (wt/vol) uranyl formate solution as previously described (48). Negative-stain micrographs were recorded manually on a JEM-2100Plus transmission electron microscope (JEOL) operated at 200 kV and equipped with a XAROSA CMOS 20-megapixel camera (EMSIS) at a nominal magnification of ×30,000 (3.12 Å per pixel).

For cryo-EM, 3 µL of 0.5 mg/mL of freshly purified ChlI with the addition of 10 mM ATP, 50 mM MgCl_2_, 0.05% lauryl maltose neopentyl glycol (LMNG), and 1 mM dithiothreitol (DTT) were applied to glow-discharged C-Flat grids (R1.2/1.3 3Cu-50) (EMS) and immediately plunge frozen in liquid ethane using a Vitrobot Mark IV (Thermo Fisher Scientific) with the environmental chamber set at 100% humidity and 4°C.

Two data sets of 4,191 and 3,495 movies were recorded automatically with EPU (Thermo Fisher Scientific) using a Glacios cryo-transmission electron microscope (Thermo Fisher Scientific) operated at 200 kV and equipped with a Selectris energy filter and a Falcon 4 detector (both Thermo Fisher Scientific). Data were recorded in electron event representation (EER) mode at a nominal magnification of ×130,000 (0.924 Å per pixel) in the defocus range of −0.8 to −1.8 µm with an exposure time of 7.50 s, resulting in a total electron dose of approximately 50 e^−^ Å^−2^.

### Cryo-EM image processing

All cryo-EM data were preprocessed in cryoSPARC Live, and further processing was performed in cryoSPARC v3 and v4 (26) (Fig. S1). For all collected movies (Fig. S2A), motion correction (EER upsampling factor 1, EER number of fractions 40) and contrast transfer function (CTF) estimation were performed using cryoSPARC Live implementations. Both collected data sets were preprocessed in a similar manner and combined at the final image processing steps (Fig. S1). For the data set of 4,191 micrographs, particles were selected by the template picker implemented in CryoSPARC Live, using well-defined 2D classes obtained from previous ChlI data sets as templates. Picked particles were extracted in a box size of 288 pixels and Fourier cropped to 144 pixels (resulting in a pixel size of 1.848 Å per pixel) and subjected to 2D classification, which produced 2D classes that were used as templates for another round of template particle picking in CryoSPARC, followed by particle extraction using the same box size of 288 pixels (Fourier cropped to 144 pixels) and several rounds of 2D classification (Fig. S2B) to eliminate bad picks. In addition, particles were picked using the Topaz (49) wrapper in CryoSPARC and extracted using a box size of 288 pixels (Fourier cropped to 144 pixels). All picked particles were then combined, and duplicates were removed, resulting in a stack of 581.4K particles. These particles were then subjected to two rounds of 2D classification. The 3,495-movie data set was processed similarly, and all particle pickers applied there resulted in a stack of 375.2K particles after duplicate removal (Fig. S1). These particles were also subjected to two rounds of 2D classification.

Particles from the two data sets were then combined and used for four rounds of *ab initio* reconstructions with multiple classes, followed by heterogeneous refinements. The two best hexamer classes (80.5K and 58.3K particles) and the class corresponding to the pentamer (24.7K particles) from the last round of heterogeneous refinement were further refined by non-uniform refinement (50) using particles re-extracted with the full box size (288 pixels, 0.924 Å per pixel), resulting in reconstructions with resolutions of 3.9 Å and 4.1 Å for the hexamers and 5.6 Å for the pentamer (gold standard Fourier shell correlation [GSFSC] value of 0.143]). Consequently, the reconstructions were further improved using the non-uniform refinement with CTF refinement on the fly, resulting in consensus maps with resolutions of 3.8 Å and 4 Å for the hexamers and 4.9 Å for the pentamer (GSFSC = 0.143) (Fig. S1).

All maps were subjected to unsupervised B-factor sharpening within cryoSPARC. No symmetry was applied during processing. The quality of the consensus maps is shown in Fig. S2. All GSFSC curves were generated within cryoSPARC. The local resolutions of the consensus maps (Fig. S2) were estimated in cryoSPARC and analyzed in UCSF ChimeraX (51). Data set statistics are provided in Table S1.

### Model building and refinement

For each of the three structures, the same approach has been used to build the model. The AlphaFold structure prediction of the ChlI monomer (AlphaFold accession: AF-P58571-F1; Uniprot: P58571) was manually fitted to each of three maps of ChlI using the “Fit in Map” tool in ChimeraX (51) and used as a starting model. The N-terminal (residues 1–13) and C-terminal (residues 354–374) fragments of ChlI without well-resolved densities were removed. The models of the ChlI monomer were then manually adjusted and refined in Coot (52). The models of the other monomers of ChlI were generated based on the built structure of the first monomer and fitted to the ChlI cryo-EM maps using UCSF ChimeraX. Models of individual monomers were then combined into a single structure and manually inspected and refined in Coot, and models of respective nucleotides and magnesium ions were fitted inside the corresponding densities. The fragments of ChlI monomers with missing corresponding densities in the cryo-EM maps were manually deleted in Coot. Subsequently, iterative rounds of real-space refinement (53) of the models against the corresponding ChlI maps in PHENIX (54) were performed, followed by manual adjustments in Coot. The model of the ChlI pentamer was truncated to poly-alanine using the PDB Tools job in PHENIX. Model validation was done using MolProbity (55) in PHENIX. Models and maps were visualized, and figures were prepared in UCSF ChimeraX and Inkscape. Model refinement and validation statistics are provided in Table S1.

## Data Availability

The cryo-EM density maps and corresponding atomic models reported in this study have been deposited in the Electron Microscopy Data Bank and Protein Data Bank with the accession codes EMD-17151, EMD-17152, EMD-17153, and PDB-8OSF, PDB-8OSG, and PDB-8OSH, respectively.

## SUPPLEMENTAL MATERIAL

Supplemental Material mbio.01893-23-s0001.docx Table S1, movie captions, and Figures S1-S4.

Movie S1 mbio.01893-23-s0002.mp4 ChlI hexamer conformation A.

Movie S2 mbio.01893-23-s0003.mp4 ChlI hexamer conformation B.

Movie S3 mbio.01893-23-s0004.mp4 Morph of hexamer conformation A to hexamer conformation B.

ASM does not own the copyrights to Supplemental Material that may be linked to, or accessed through, an article. The authors have granted ASM a non-exclusive, world-wide license to publish the Supplemental Material files. Please contact the corresponding author directly for reuse.
