# Supplementary material for: Conformational variability of cyanobacterial ChlI, the AAA+ motor of magnesium chelatase involved in chlorophyll biosynthesis
Source: mBio. 2023 Sep 22;14(5):e01893-23. doi: 10.1128/mbio.01893-23 (PMC10653834; doi:10.1128/mbio.01893-23)
Supplement: Supplemental Material — Table S1, movie captions, and Figures S1-S4. [file mbio.01893-23-s0001.docx]

**Supplementary Information**

**Table S1. Cryo-EM data collection, refinement and validation statistics**

|  | Hexamer conformation A  (EMDB-17151)  (PDB 8OSF) | Hexamer conformation B  (EMDB-17152)  (PDB 8OSG) | Pentamer conformation  (EMDB-17153)  (PDB 8OSH) |
| --- | --- | --- | --- |
| **Data collection and processing** |  |  |  |
| Magnification | 130,000 | 130,000 | 130,000 |
| Voltage (kV) | 200 | 200 | 200 |
| Electron exposure (e–/Å^2^) | 50 | 50 | 50 |
| Defocus range (μm) | -0.8 to -2.8 | -0.8 to -2.8 | -0.8 to -2.8 |
| Pixel size (Å) | 0.924 | 0.924 | 0.924 |
| Symmetry imposed | C1 | C1 | C1 |
| Initial particle images (no.) | 4,940,012 | 4,940,012 | 4,940,012 |
| Final particle images (no.) | 58,137 | 80,344 | 24,589 |
| Map resolution (Å)  FSC threshold | 4.0  0.143 | 3.8  0.143 | 4.9  0.143 |
|  |  |  |  |
| **Refinement** |  |  |  |
| Initial model used | AlphaFold | AlphaFold | AlphaFold |
| Model resolution range (Å)  FSC threshold | 3.0-6.0  0.143 | 3.0-6.0  0.143 | 4.0-7.0  0.143 |
| Map sharpening *B* factor (Å^2^) | -103.3 | -105.8 | -189.5 |
| Model composition  Non-hydrogen atoms  Protein residues  Ligands | 14529  1829  MG (5), ATP (5), ADP (1) | 14536  1833  MG (4), ATP (4), ADP (2) | 6770  1372  - |
| R.m.s. deviations  Bond lengths (Å)  Bond angles (°) | 0.002  0.690 | 0.004  0.743 | 0.006  0.942 |
| Validation  MolProbity score  Clashscore  Poor rotamers (%) | 2.15  12.05  0.56 | 2.00  9.70  0.19 | 1.90  5.83  0 |
| Ramachandran plot  Favored (%)  Allowed (%)  Outliers (%) | 89.68  10.32  0 | 91.84  8.11  0.06 | 88.72  11.20  0.08 |
|  |  |  |  |

**Movie Captions**

**Movie S1.** Movie representation of the hexamer conformation A of ChlI, as determined by cryo-EM. Data is represented as cryo-EM density and corresponding molecular model. Coloring as in Figure 2. Bound nucleotides and structured insertions are indicated.

**Movie S2.** Movie representation of the hexamer conformation B of ChlI, as determined by cryo-EM. Data is represented as cryo-EM density and corresponding molecular model. Coloring as in Figure 2. Bound nucleotides and structured insertions are indicated.

**Movie S3.** Conformational changes of ChlI. A morph representation of differences between conformation A and B. Coloring as in Figure 2.


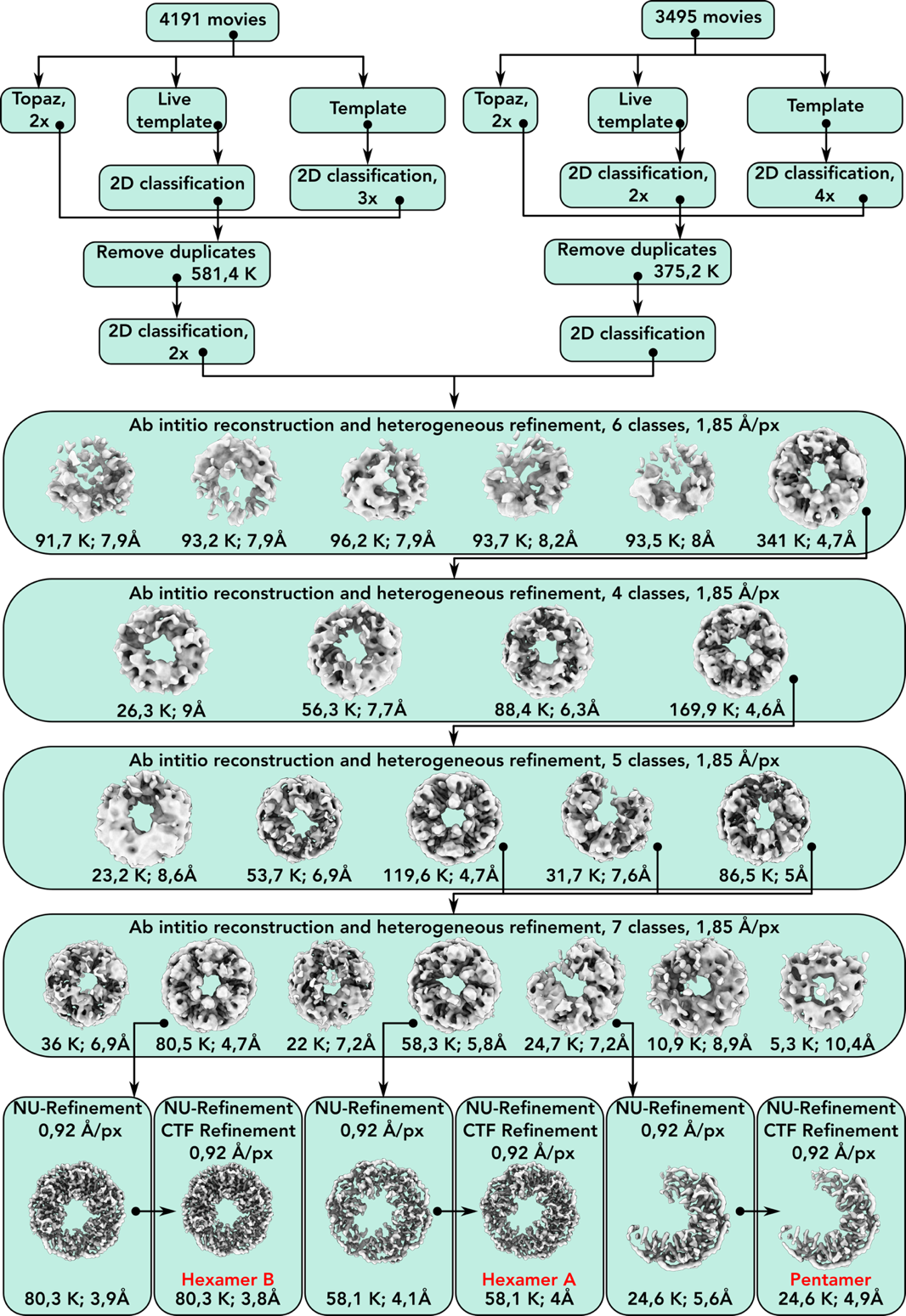


**Figure S1.** Scheme of cryo-EM image processing. A detailed description of the data analysis can be found in the Materials and Methods section. The entire pipeline was performed in CryoSPARC v.3 and v.4. Briefly, two datasets were collected. After preprocessing in cryoSPARC Live, particles were picked using the template and Topaz pickers. For the template picker, 2D classes from our trial attempts of ChlI cryo-EM analysis were used as templates. After particle picking, the particles were 2D classified, and duplicate particles were removed. Next, additional 2D classification was performed, and the retained particles were combined and subjected to several rounds of ab initio reconstruction and heterogeneous refinement with multiple classes. Particles from the two best ring classes and the pentamer class from the last round of heterogeneous refinement were separately re-extracted using a full box (0.92 Å/pixel) and subjected to a round of NU-refinement for each class. Another round of NU-refinement with CTF refinement was used to further improve the maps. The final maps achieved resolutions of 3.8 Å, 4 Å (for hexamers) and 4.9 Å (for pentamer). No symmetry was applied at any stage of the processing pipeline.


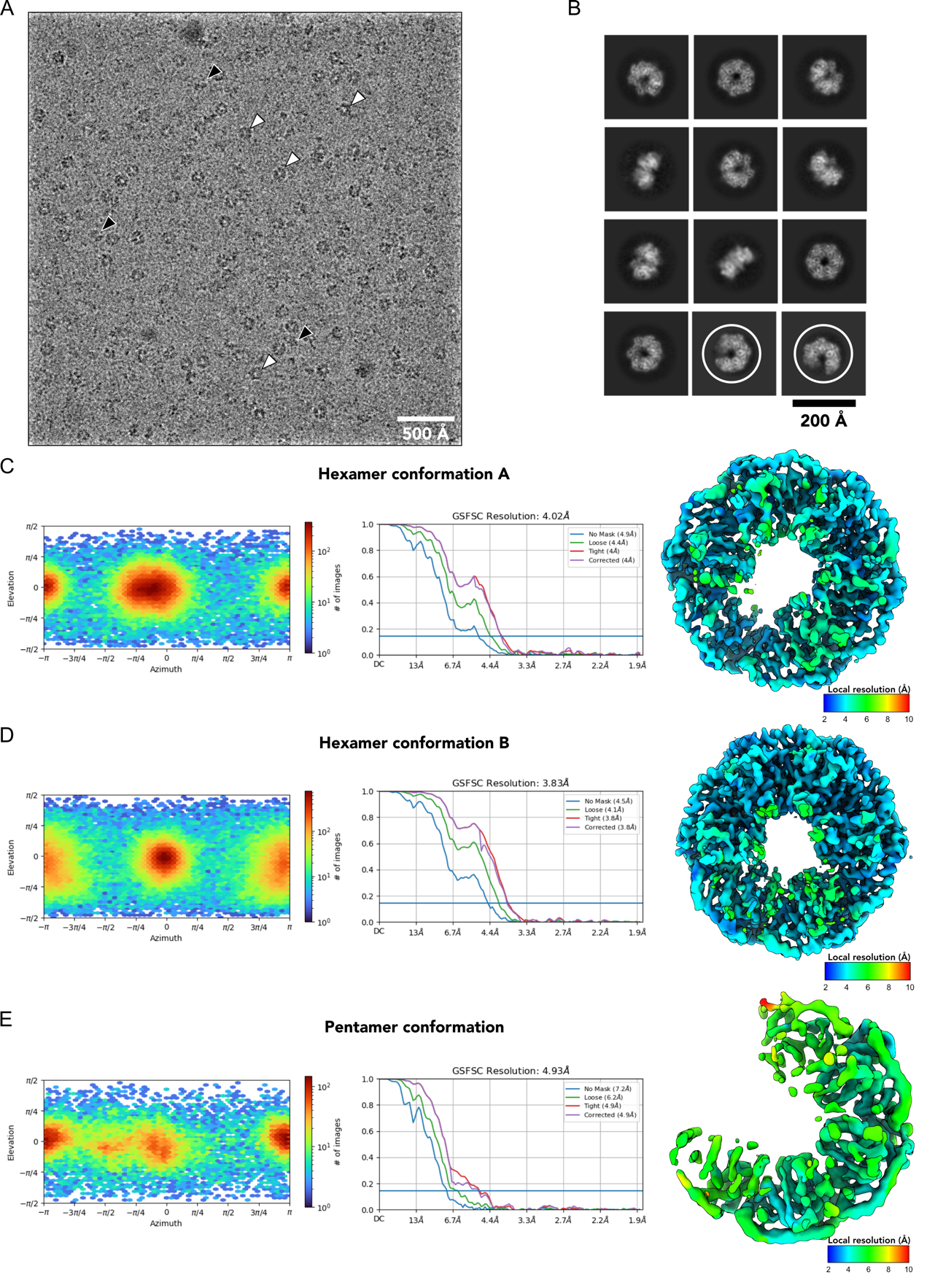


**Figure S2.** A) Representative cryo-EM micrograph showing ChlI particles indicated viewed from above (white triangles) or from the side (black triangles). B) representative 2D class averages showing different view projections of ChlI. White circles indicate 2D classes corresponding to the pentamer conformation. (C-E) Validation of ChlI cryo-EM structures. Data for the hexamer conformation A (C), hexamer conformation B (D), and pentamer conformation (E). In each panel (C-E), the the angular distribution plot of the particles used for the reconstruction is shown in the left, Gold Standard Fourier Shell Correlation (GSFSC) curve with the blue horizontal line marking the GSFSC value of 0.143 is shown in the center, and the local resolution estimate of the respective cryo-EM map, calculated in CryoSPARC (see color code bar), is shown on the right.


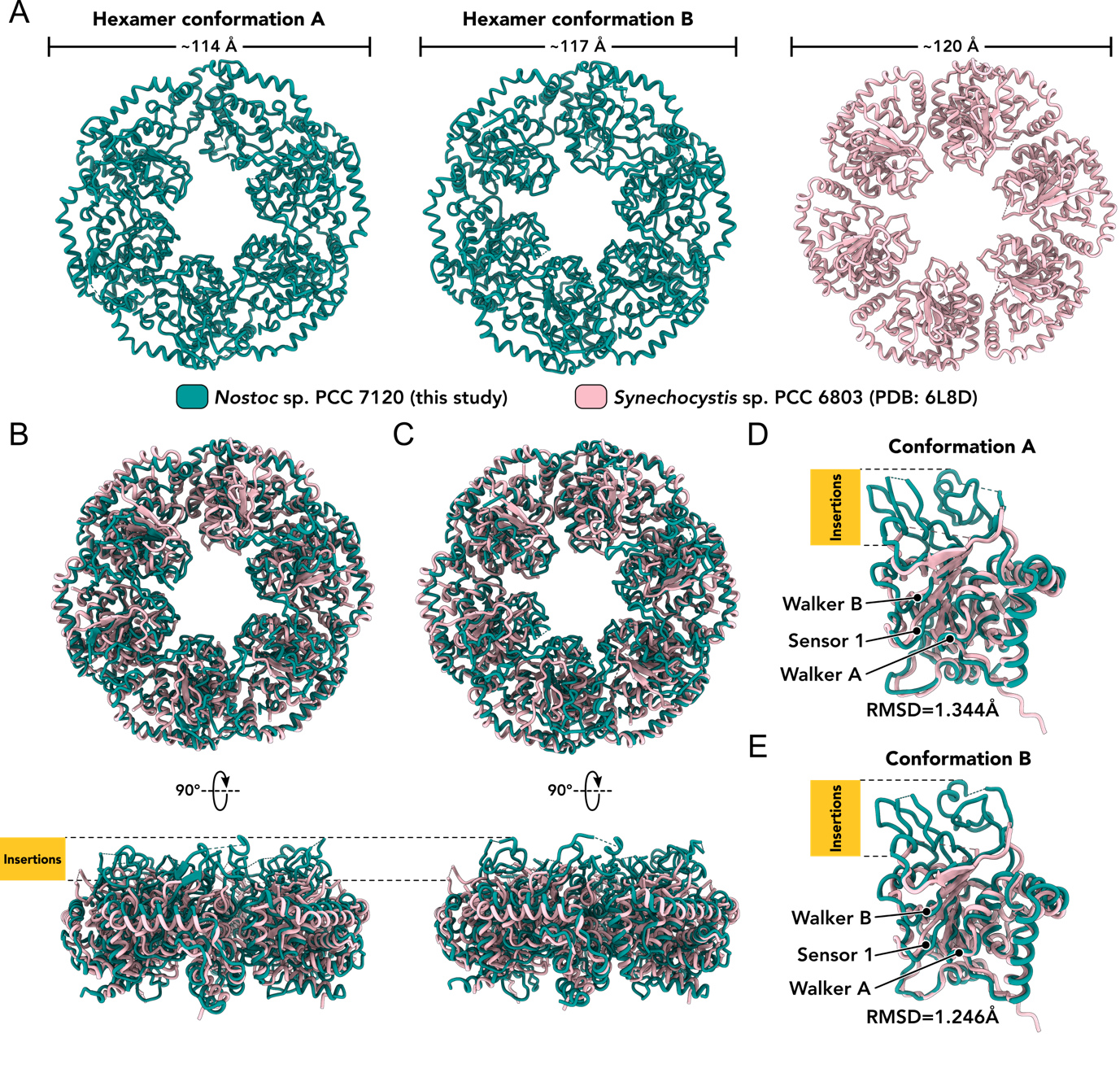


**Figure S3.** Comparison of cryo-EM hexamer structures of ChlI from *Nostoc* (this study, teal) with published X-ray ChlI structure from *Synechocysts* (PDB: 6L8D, pink). A) Ribbon representations of the *Nostoc* ChlI structures and the structure of *Synechocysts* ChlI viewed from above. Approximate ring dimensions are shown; B) Superposition of *Nostoc* ChlI conformation A with *Synechocystis* ChlI achieved by fitting the *Synechocystis* structure into the density map generated from the *Nostoc* structure using the “molmap” command in ChimeraX. Top and side views are shown and the area of structured insertions is highlighted; C) Superposition of *Nostoc* ChlI conformation B with *Synechocystis* ChlI, shown as in (B); D) Superposition of *Nostoc* ChlI monomer from conformation A with *Synechocystis* ChlI monomer using the "matchmaker" command in ChimeraX. Root-mean-square deviation (RMSD) between 189 residue pairs is indicated and the positions of the structured insertions, Walker A, Walker B, and Sensor 1 motifs are shown on the overlaid structures; E) Superposition of *Nostoc* ChlI monomer from conformation B with *Synechocystis* ChlI monomer shown as in D. RMSD corresponds to 190 residue pairs.


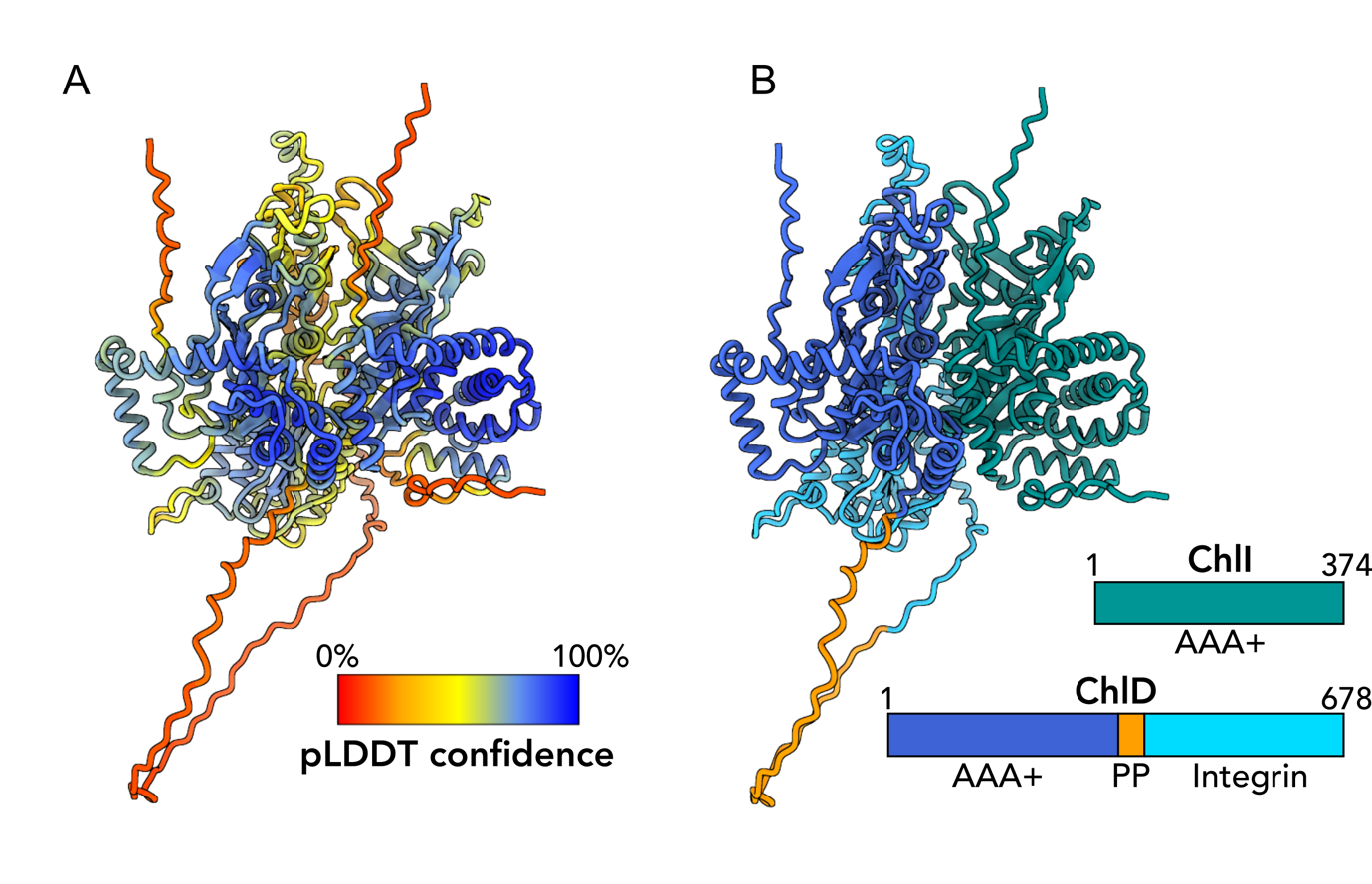


**Figure S4.** AlphaFold modeling of ChlI interactions with the partner protein ChlD. A) Ribbon representation of a ChlI-ChlD complex model colored by the pLDDT confidence of the structure prediction (see color code bar); B) Predicted complex of ChlI and ChlD viewed as in (A) and colored by protein domains. Schematic overview of ChlI and ChlD protein sequences is shown. The integrin domain (light blue) and the polyproline linker region (orange), which are characteristic for the ChlD protein, are indicated in the schemes and in the structure representation.
